# Natural SINEUP RNAs in Autism Spectrum Disorders: *RAB11B-AS1* Dysregulation in a Neuronal *CHD8* Suppression Model Leads to RAB11B Protein Increase

**DOI:** 10.3389/fgene.2021.745229

**Published:** 2021-11-22

**Authors:** Giulia Zarantonello, Michele Arnoldi, Michele Filosi, Toma Tebaldi, Giovanni Spirito, Anna Barbieri, Stefano Gustincich, Remo Sanges, Enrico Domenici, Francesca Di Leva, Marta Biagioli

**Affiliations:** ^1^ Laboratory of Neuroepigenetics, Department of Cellular, Computational and Integrative Biology (CIBIO), University of Trento, Trento, Italy; ^2^ Laboratory of Neurogenomic Biomarkers, Department of Cellular, Computational and Integrative Biology (CIBIO), University of Trento, Trento, Italy; ^3^ Section of Hematology, Yale Cancer Center and Department of Internal Medicine, Yale University School of Medicine, New Haven, United States; ^4^ Laboratory of RNA and Disease Data Science, Department of Cellular, Computational and Integrative Biology (CIBIO), University of Trento, Trento, Italy; ^5^ Laboratory of Computational Genomics, Area of Neuroscience, International School of Advanced Studies (SISSA), Trieste, Italy; ^6^ Central RNA Laboratory, Italian Institute of Technology (IIT), Genova, Italy; ^7^ Fondazione The Microsoft Research - University of Trento Centre for Computational and Systems Biology (COSBI), Rovereto, Italy

**Keywords:** autism spectrum disorders (ASD), *CHD8*, lncRNA, natural antisense transcript (NAT), SINEUP, post-transcriptional regulation, neurodeveloment, RAB11 GTPase

## Abstract

*CHD8* represents one of the highest confidence genetic risk factors implied in Autism Spectrum Disorders, with most mutations leading to *CHD8* haploinsufficiency and the insurgence of specific phenotypes, such as macrocephaly, facial dysmorphisms, intellectual disability, and gastrointestinal complaints. While extensive studies have been conducted on the possible consequences of *CHD8* suppression and protein coding RNAs dysregulation during neuronal development, the effects of transcriptional changes of long non-coding RNAs (lncRNAs) remain unclear. In this study, we focused on a peculiar class of natural antisense lncRNAs, SINEUPs, that enhance translation of a target mRNA through the activity of two RNA domains, an embedded transposable element sequence and an antisense region. By looking at dysregulated transcripts following *CHD8* knock down (KD), we first identified *RAB11B-AS1* as a potential SINEUP RNA for its domain configuration. Then we demonstrated that such lncRNA is able to increase endogenous RAB11B protein amounts without affecting its transcriptional levels. RAB11B has a pivotal role in vesicular trafficking, and mutations on this gene correlate with intellectual disability and microcephaly. Thus, our study discloses an additional layer of molecular regulation which is altered by *CHD8* suppression. This represents the first experimental confirmation that naturally occurring SINEUP could be involved in ASD pathogenesis and underscores the importance of dysregulation of functional lncRNAs in neurodevelopment.

## Introduction

Autism Spectrum Disorders (ASD) are a heterogeneous group of complex neurodevelopmental conditions characterized by social-communicative deficits as well as repetitive sensory-motor behaviors, appearing during early childhood (American Psychiatric Association, 2013). ASD prevalence is steadily increasing, such that the estimated global prevalence is currently 1 in 68 (Elsabbagh et al., 2012). Adding to complexity, prevalence in males is 4 to 5-fold higher than in females (Baio et al., 2018). Albeit affecting such a significant portion of the world population, the underlying mechanisms of the disease have not yet been elucidated. However, several etiological hypotheses have been proposed, with risk factors ranging from environmental, to epigenetic (Shulha et al., 2012; Ladd-Acosta et al., 2014), to genetic. Consistently with clinical heterogeneity, the genetic architecture of ASD includes variable inheritance patterns, including rare *de novo* variants, chromosomal alterations, and common inherited variation (De Rubeis and Buxbaum, 2015; de la Torre-Ubieta et al., 2016). To date, more than 1,000 genes have been ranked as potential risk factors for ASD [SFARI Gene (Abrahams et al., 2013)], and it is challenging to determine whether they converge on shared molecular mechanisms.

Major efforts in transcriptomics profiling are proposing a unifying model (Gandal et al., 2018), advancing the hypothesis that convergent molecular abnormalities are identifiable in autistic brains (Voineagu et al., 2011). Aberrant transcription remains a prevalent feature in ASD (Voineagu et al., 2011). Consistently with the strong impact on the transcriptome, many ASD-linked genes are chromatin modifiers and transcriptional regulators. Among them, Chromodomain Helicase DNA-Binding protein 8 (*CHD8*) is currently one of the highest confidence risk factors (Satterstrom et al., 2020), with *de novo* haploinsufficiency leading to a genetically defined ASD subtype, characterized by distinctive facial dysmorphisms, macrocephaly (Bernier et al., 2014), mild intellectual disability and postnatal overgrowth (Ostrowski et al., 2019). From a molecular standpoint, *CHD8* suppression leads to changes in epigenetic marks, splicing aberrations and broad transcriptional dysregulation, impacting the coding, and non-coding transcriptome, as confirmed in independent RNA-seq studies on human neuronal models (Sugathan et al., 2014; Cotney et al., 2015; Wang et al., 2015; Wilkinson et al., 2015), cerebral organoids (Wang et al., 2017) and mouse models (Durak et al., 2016; Katayama et al., 2016; Gompers et al., 2017; Platt et al., 2017; Jung et al., 2018; Suetterlin et al., 2018). Typically, changes in coding genes expression could be directly linked to cellular pathways and biological functions, such as cell cycle, Wnt signalling, RNA transcriptional regulation and chromatin remodelling, as well as cancer-related genes, other ASD risk genes, and neural development-relevant genes (Sugathan et al., 2014; Cotney et al., 2015; Wang et al., 2015; Wilkinson et al., 2015; Durak et al., 2016; Katayama et al., 2016; Gompers et al., 2017; Platt et al., 2017; Wang et al., 2017; Jung et al., 2018; Suetterlin et al., 2018). Conversely, lncRNAs play a major role in neural functioning, development and brain disorders (van de Vondervoort et al., 2013; Chen and Zhao, 2014; Hosseini et al., 2019) and they were found broadly dysregulated in post-mortem brain samples from ASD patients (Ziats and Rennert, 2013; Parikshak et al., 2016). However, the functional implication of lncRNAs in ASD biology remains vastly unclear. These lines of evidence call for further studies about the roles of lncRNAs in nervous system pathology.

Therefore, we decided to investigate the potential presence of functional lncRNAs among the dysregulated genes in *CHD8* suppression Human induced Neural Progenitor Cells (hiNPCs) model, hypothesizing that they may constitute a further layer of molecular regulation in ASD. However, *in silico* prediction of lncRNAs functionality is intrinsically challenging, as non-coding transcripts only rarely have a modular structure, therefore structure-to-function relationships are not always straightforward (Mattick, 2018). SINEUP is a novel class of functional antisense lncRNA, which can up-regulate protein translation of their target sense mRNAs, without altering their transcription (Zucchelli et al., 2015a; Zucchelli et al., 2015b). First discovered in mouse (Carrieri et al., 2012), where *Uchl1-AS* was found to up-regulate protein translation of *Uchl1* mRNA, SINEUP translational increase is mediated by two functional domains*,* namely 1) a region overlapping the Translational Initiation Site (TIS), head-to-head antisense to the 5′ end of the target sense mRNA, which confers specificity to the protein coding transcript (binding domain, BD) and 2) a SINEB2 repeat, Alu, MIR transposable element (TE) (Carrieri et al., 2012; Patrucco et al., 2015; Zucchelli et al., 2015b; Schein et al., 2016) on the 3′ end, that mediates the effect on the target mRNA translation (effector domain, ED). Several transcripts with this structure were computationally identified, and their function as SINEUPs successfully confirmed (Carrieri et al., 2012; Schein et al., 2016). Thus, it is possible to confidently hypothesize the function of such lncRNAs based merely on their structure.

In this work, we sought to identify SINEUP-like molecules among the dysregulated transcripts in an ASD-relevant cellular model system, human neural progenitors where *CHD8* expression was suppressed by approximately 50% using short hairpin RNAs (shRNAs), mimicking the haploinsufficiency condition (Sugathan et al., 2014). Among the identified candidates, we prioritized the *RAB11B-AS1* lncRNA, and provided experimental evidence of its regulatory role on its sense counterpart *RAB11B* mRNA by means of its SINEUP-specific domains. Our results suggest that ASD transcriptional dysregulation might affect previously unrecognized lncRNAs-mediated networks and underline SINEUP molecules as unacknowledged players in ASD molecular phenotypes.

## Materials and Methods

### Human Induced Neural Progenitor Cells Culture

hiNPCs from fibroblasts of a control individual, GM8330-8 (Sheridan et al., 2011), were used to generate stable KD lines, where shRNAs targeting *CHD8 (sh4, sh2, sh1)* or GFP as control (shGFP) were delivered (Sugathan et al., 2014). Cells were cultured on poly-L-ornithine hydrobromide (20 μg/ml, #P3655 Sigma)/laminin (3 μg/ml, #23017015 Life Technologies)–coated plates in hiNPC medium [70% v/v DMEM (Life Technologies) completed with 30% v/v HAM F12 (#ECB7502L, Euroclone), 2% v/v B27 (#17504001, Life Technologies), 1% v/v Penicillin-Streptomycin solution (#15140122, Life Technologies), 1% v/v L-Glutamine (#25-005-CI, Corning), supplemented with EGF (20 ng/ml, #E9644, Sigma), bFGF (20 ng/ml, #233-FB R and D), Heparin (5 μg/ml, #H9267 Sigma)]. Semi-confluent monolayers were maintained in 5% CO_2_, 37°C humidified incubator.

### Identification of Candidate SINEUP

**FIGURE 1:**
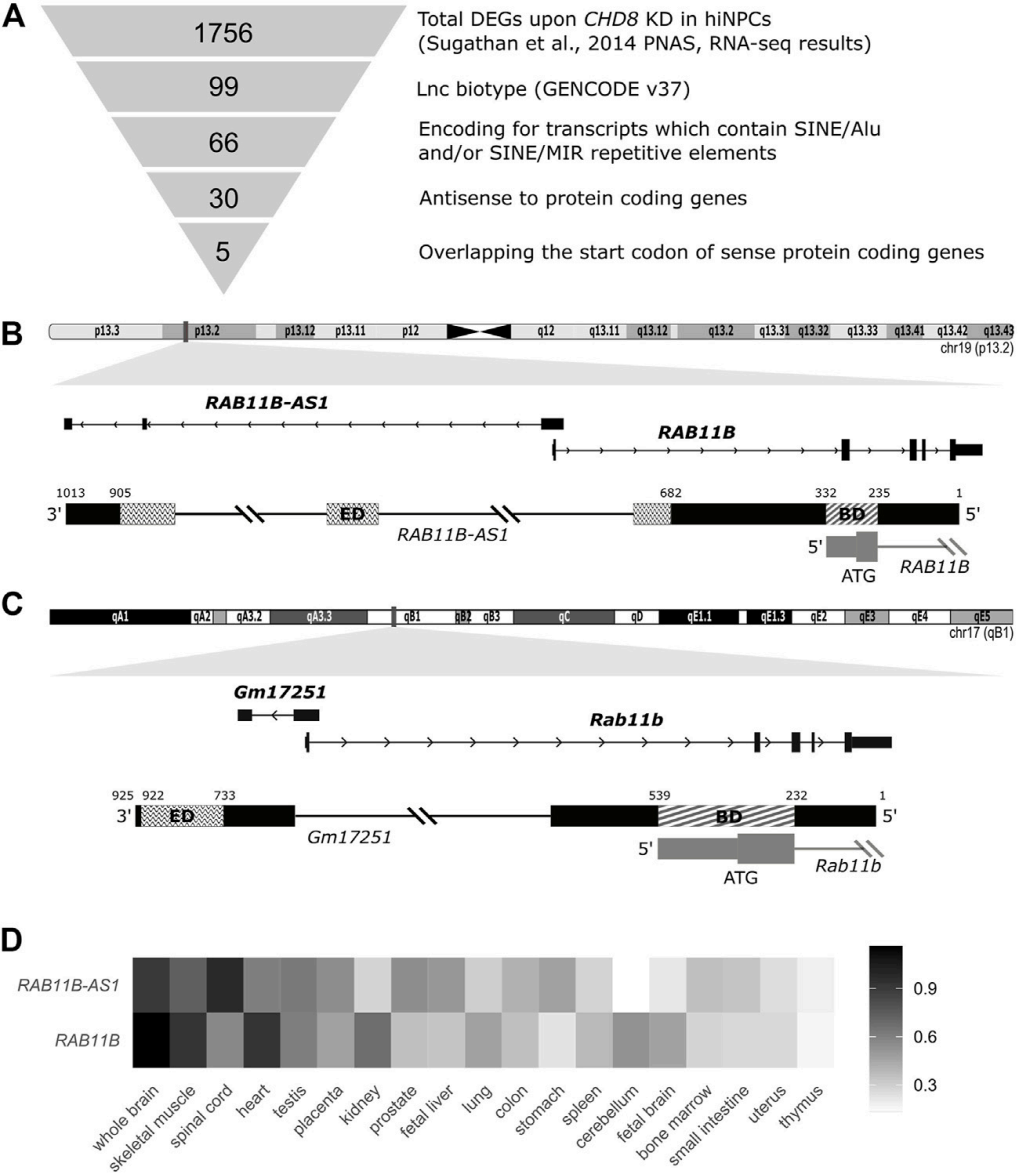
Identification of SINEUP-like molecules among deregulated transcripts following *CHD8* suppression. **(A)** Schematic representation of the employed pipeline, with sequential filtering steps for the selection of candidate SINEUP-like molecules. **(B)** Integrative Genome Viewer (IGV) view of the candidate antisense lncRNA *RAB11B-AS1* and the respective sense counterpart coding mRNA *RAB11B* (Human RefSeq annotation). Putative SINEUP functional domains are highlighted (Binding Domain, BD) (Effector Domain, ED). **(C)** IGV track for M. musculus *RAB11B-AS1/RAB11B* transcripts orthologues, respectively, *Gm17251* and *Rab11b*. Putative functional domains are conserved and highlighted (BD) (ED). **(D)** Heatmap depicts normalized expression levels (2^−∆∆Cq^) of *RAB11B* and *RAB11B-AS1*, illustrating concordant expression across human tissues, with highest levels reported in black and lowest in white.

SINEUP-like transcripts were identified among the Differentially Expressed Genes (DEGs) comparing WT and *CHD8* KD hiNPCs (GSE61491, GEO, NCBI) (Sugathan et al., 2014) according to a series of filtering steps (Figure 1A): 1) LncRNAs were selected relying on GENCODE v37 lncRNA gene annotation (Frankish et al., 2019); 2) Screening for SINE/Alu or SINE/MIR TEs was performed using Dfam Tool Repeat Masker v3.0 (Smit et al., 1996); 3) Antisense overlapping transcripts to sense coding mRNAs were identified using the BioConductor package GenomicRanges (Lawrence et al., 2013) (minoverlap = 1 L); 4) Transcripts overlapping the respective sense protein coding gene on the first ATG were chosen by comparing the “start codon” position of the sense mRNA to exons “start” and “end”positions of the lncRNAs from GENCODE v37 comprehensive annotation, and confirmed by using Ensembl (Yates et al., 2020) and UCSC Genome Browser (Kent et al., 2002) annotations.

### RNA Extraction and Retrotranscription

Total RNA was extracted using TRIzol (#15596018 Ambion, Life Technologies) following the manufacturer’s instructions. Genomic DNA was removed using DNase I (#AM2222 Ambion, Life Technologies) incubation (0.2–1 µg RNA with 2U of DNaseI) in DNase Buffer with 1U of RNase inhibitor (#AM2684, Ambion, Life Technologies), for 30′, at 37°C. Treated RNA samples were purified with RNeasy Mini Kit (#74104, QIAGEN). Reverse transcription was performed using SensiFAST™ cDNA synthesis kit (#BIO65053, Bioline) with Oligo-dT/random hexamers primers according to manufacturer’s instructions. cDNA diluted 1:10 was used for qPCR. Transcripts relative expression levels in human tissues were determined using Human Total RNA Master Panel (#636643, LOT1409502A, ClonTech). 1 µg was retro-transcribed for each tissue/cell type as previously described, and cDNA was diluted 1:20 for application in qPCR.

### Quantitative PCR

Primers for qPCR were designed spanning an exon-exon junction by using the Universal Probe Library Assay Design Center (Roche Life Science, 2019). iTaq™ Universal SYBR® Green Supermix (#1725121, Biorad) was used following manufacturer’s instructions. *NONO* reference gene (Eisenberg and Levanon, 2013) was used for normalization, and relative expression values were calculated using the 2^−∆∆Cq^ method (Segundo-Val and Sanz-Lozano, 2016). The co-expression pattern of S/AS pairs across Human Total RNA Master Panel was evaluated by plotting the normalized and relativized expression values (2^−∆∆Cq^) matrix into a heatmap. In this case, for each gene, the ∆∆Cq ratio was calculated with respect to the highest expression value across tissues. Amplicons size and specificity were verified through gel electrophoresis and Sanger sequencing.

### Cloning

To clone *RAB11B-AS1* we performed gene-specific (GS) retrotranscription from GM8330-8 total RNA, using RevertAid First Strand cDNA Synthesis Kit (#K1622, Thermo Fisher) according to the manufacturer’s instructions. 1 µg of total RNA was retrotranscribed with the GS primer h*RAB11B-AS1*-GS (5′-TCT​TTA​GTT​CAC​AGA​TCT​AGT​A-3′). Primers to clone *RAB11B-AS1* and *RAB11B* were designed on the transcript 5′ and 3′ ends, and restriction sites were added for ligation. PCR to amplify *RAB11B* and *RAB11B-AS1* was performed using Phusion Green Hot Start II High-Fidelity PCR Master Mix (#F566S, Thermo Fisher). pcDNA™ 3.1 (-) vector (#V795-20, Invitrogen) was the backbone to clone full-length *RAB11B-AS1* sequence between EcoRI, HindIII restriction sites [pcDNA 3.1 (-)-h*RAB11B-AS1*-WT]. Domain-targeted deletion mutants were created by using the Q5 R Site-Directed Mutagenesis kit (#E0554, NEB) according to manufacturer’s instructions. Primers for *RAB11B-AS1* mutagenesis were generated using NEBase Changer™ v1.2.9 web tool (NEBaseChanger, 2019). After transformation, positive clones were Sanger sequenced to verify the proper insertion of the sequence and/or effective deletion of target domains.

### hiNPCs Electroporation

Roughly 5·10^6^ GM8330-8 hiNPCs were electroporated in 100 µl of electroporation solution (5 mM KCl, 15 mM MgCl_2_, 10 mM C_6_H_12_O_6_, and 120 mM K_2_HPO_4_/KH_2_PO_4_ 1 M pH 7.2) with 5 µg of plasmid using program A-033 of the Amaxa Nucleofector™ 2b Device (#AAB1001, Lonza). After electroporation, cells were directly resuspended in hiNPCs complete medium and plated on poly-ornithine/laminin coated dishes. Cells were eventually harvested for subsequent analysis after 48 h.

### Protein Extraction and Western Blot

Total proteins were extracted in RIPA Buffer (#R0278, Sigma-Aldrich), with Protease Inhibitor (#88266, Thermo Fisher). After sonication (Q700, Qsonica) and centrifugation at 12.000 rpm for 20′ at 4°C, the supernatant was quantified using bicinchoninic acid (BCA) protein quantification (#23225, Thermo Fisher) following the manufacturer’s instructions. 8–15 µg of proteins were run on NuPAGE™ 4–12% Bis-Tris Protein Gel (#NP322, Invitrogen) in MOPS SDS Running Buffer (#NP0001, Novex, Life Technologies) for 2 h, 120 V. Transfer was carried out on Polyvinylidene Fluoride (PVDF) membrane in Tris-Glycine Buffer (#28363, Thermo Fisher) with 5% methanol, at 70 V for 30′ at 4°C. Membranes were blocked with 5% non-fat milk in PBS-Tween (0.1%) at room temperature (RT) and incubated with rabbit anti-RAB11B 1:1000 (#orb30974, Biorbyt or #HPA054396, Atlas Antibodies) or mouse anti-β-tubulin 1:5000 (#sc-53140, Santa Cruz Biotechnology) primary antibodies overnight at 4°C. After three washes with PBS-Tween (0.1%), membranes were incubated with HRP conjugated goat anti-rabbit (#074-1506, KPL) or goat anti-mouse (#5220-0341, KPL) secondary antibodies (1:5000), for 1 h at RT. The membrane was developed with ECL solution (#RPN2235, GE Healthcare or #EMP011005, EuroClone) using BioRad Chemidoc XRS + System. Bands analysis was performed using ImageJ-1.53a.

### Statistical Analysis

Statistical analysis tests were performed using R as described in figure captions. Significance level was set to 0.05. Data were plotted using R (ggplot2) and represented as Mean ± Standard Error of the Mean (SEM), as specified in figure legends with sample sizes. The significance level was reported as NS *p* > 0.05, *p ≤ 0.05, **p ≤ 0.01, ***p ≤ 0.001.

## Results

### Identification of *RAB11B-AS1* as a SINEUP-like Transcript Dysregulated Upon *CHD8* Suppression

To select functional lncRNAs altered following *CHD8* suppression, we applied the selection pipeline schematized in Figure 1A and detailed in Methods. We first resorted to the complete list of dysregulated genes from Sugathan et al., 2014, where 1756 DEGs were reported following *CHD8* KD compared to control hiNPCs (shGFP). Selection of natural SINEUP molecules was performed based on their specific structural criteria 1) annotation as lncRNAs, 2) presence of a SINE/Alu and/or SINE/MIR TE, 3) antisense to protein coding genes, 4) overlapping the start codon of coding gene). These sequential filtering steps (Figure 1A and Methods) led to the isolation of five candidate lncRNA genes (Table 1) containing at least one inverted SINE/Alu and/or SINE/MIR repeats and overlapping in antisense orientation to the TIS of their respective sense protein coding mRNA. *RAB11B-AS1* (ENSG00000269386) was identified as the most promising candidate, since the structure of the transcript precisely mirrored the one of a canonical SINEUP molecule (Figure 1B). *RAB11B-AS1* transcript is the antisense counterpart of a sense, protein coding gene, *RAB11B* (ENSG00000185236), a small GTPase belonging to the Ras superfamily responsible for vesicle formation, transport, and fusion (Stenmark and Olkkonen, 2001). RAB11B is enriched in the brain (Lai et al., 1994), and it is involved in membrane and vesicle trafficking and apical proteins recycling (Kelly et al., 2012), processes of relevance for brain development and synaptic plasticity (Villarroel‐Campos et al., 2014). The *RAB11B* S/AS pairs were both significantly up-regulated upon *CHD8* suppression in hiNPCs (Table 1). Notably, another previously generated and independently characterized sh-*CHD8* suppression model (Cotney et al., 2015) displayed similar *RAB11B-AS1* upregulation, although not nominally statistically significant after multiple test correction.

**TABLE 1:** Candidate SINEUP-like transcripts dysregulated upon *CHD8* KD in hiNPCs.

LncRNA SINEUP-like gene candidates						Sense mRNA genes					
Gene name	EnsID	FC	Pvalue	Reg. Direction	Bound/Unbound	Gene name	EnsID	FC	Pvalue	Reg. Direction	Bound/Unbound
*RP11-400F19.6*	ENSG00000266962	2.03495	0.00009	up	0	*HSD17B1*	ENSG00000108786	1.29275	0.60422	noreg	0
*RP11-115C21.2*	ENSG00000246089	1.89649	0.00336	up	0	*MCPH1*	ENSG00000147316	1.12372	0.48333	noreg	1
*RAB11B-AS1*	ENSG00000269386	1.84850	0.00519	up	0	*RAB11B*	ENSG00000185236	1.46253	0.02023	up	1
*ST7-AS1*	ENSG00000227199	-1.76710	0.00991	down	0	*ST7*	ENSG00000004866	1.06260	0.71902	noreg	1
*CTD-2517M22.14*	ENSG00000255182	2.60614	0.02712	up	0	*PPP1R16A*	ENSG00000160972	2.17035	0.00004	up	0

The fold change (FC) levels with respect to the control line are indicated for the antisense and the corresponding sense coding transcripts. *p*-value and the direction of regulation (up, down or no regulation) are indicated. Genes loci directly bound by CHD8 (bound) or not bound (unbound) are reported (Sugathan et al., 2014).

*RAB11B-AS1* overlaps in opposite orientation with *RAB11B*, specifically with 96 nucleotides encompassing the TIS, representing the putative BD. As for the ED, *RAB11B-AS1* contains two classes of partially overlapping inverted embedded TE, a FRAM repeat (free right arm monomer) and a SINE/Alu repeat. Because SINE/Alu might arise from dimerization of two different REs (Mighell et al., 1997), the 2 TEs were jointly considered as the potential *RAB11B-AS1* ED, a 222 nucleotides long region near the 3′ end of the transcript (Figure 1B). Significantly, an ortholog transcript in *M. musculus*, *Gm17251* (ENSMUSG00000090952), was identified, displaying a high sequence similarity [83% identity score, BLAST (Nucleotide BLAST, 2019)] to the human counterpart (Figure 1C). Equivalently to the human transcript, it possesses the SINEUP-like putative BD, overlapping with the sense *Rab11b* (ENSMUSG00000077450) on the TIS, and a putative ED consisting of a SINEB2 repeat in inverted configuration (Figure 1C). Because co-expression of the transcripts pair is essential to SINEUP protein translation function, the spatio-temporal co-expression of *RAB11B* and *RAB11B-AS1* S/AS pair was examined. *RAB11B* and *RAB11B-AS1* transcripts levels were quantified across an RNA panel from various human body districts. *RAB11B-AS1* showed a fairly ubiquitous distribution, with detectable levels in skeletal muscle, testis and heart and highest expression in the central nervous system (spinal cord, whole brain) (Figure 1D). Importantly, *RAB11B-AS1* and *RAB11B* displayed a concordant expression pattern primarily in whole brain, heart, and skeletal muscle (Figure 1D), thus supporting a possible S/AS functional regulatory mechanism.

Importantly, linear regression analysis performed on publicly available CAGE (Cap Analysis of Gene Expression) data from the FANTOM project (Severin et al., 2014; Lizio et al., 2015; Abugessaisa et al., 2021), derived from 1,886 human samples including primary cultures, tissues, and transformed cells, confirmed a positive significant correlation between *RAB11B* and *RAB11B-AS1* (R = 0.25; *p* = 3.55E-28), consistent with our results.

### *RAB11B-AS1* and *CHD8* Display Inversely Correlated Expression

**FIGURE 2:**
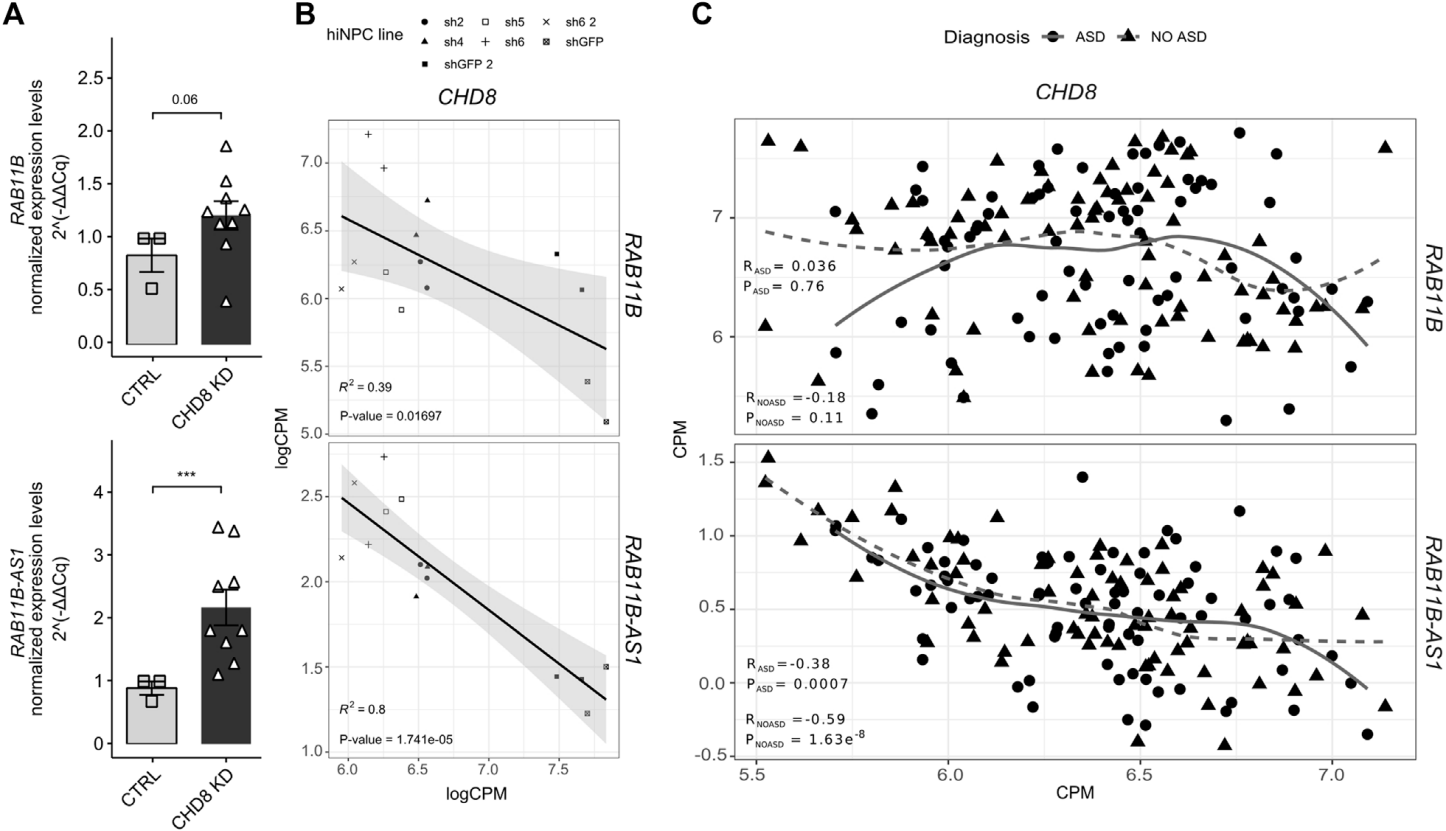
*RAB11B-AS1* and *CHD8* display inversely correlated expression. **(A)** The barplots report *RAB11B* **(top)** and *RAB11B-AS1* **(bottom)** transcripts quantification (2^−∆∆Cq^), increasing upon *CHD8* KD (average of sh4, sh2, sh1) with respect to control (shGFP line). Data are plotted as Mean ± SEM. For statistical analysis, un-paired one-tail Student’s *t*-test was performed between control and *CHD8* KD lines. N = 3 for each line. ***p ≤ 0.001. **(B)** Dot plots with linear regression line show *RAB11B* **(top)** and *RAB11B-AS1* **(bottom)** transcripts abundance (logCPM) with respect to *CHD8* levels in hiNPCs with suppressed levels of *CHD8* (Sugathan et al., 2014). Linear regression analyses unveiled a significant inverse correlation for *RAB11B* (*R* ^2^ = 0.39, *p* = 0.01697) and particularly for *RAB11B-AS1* (*R* ^2^ = 0.8, *p* = 1.741e-05) compared to *CHD8*. **(C)** Dot plots display the abundance (CPM) of *RAB11B* **(top)** and *RAB11B-AS1* **(bottom)** compared to *CHD8* from RNAseq analyses of peripheral blood samples from ASD patients (ASD diagnosis) and healthy siblings (NO ASD) in the ITAN family cohort. *RAB11B-AS1* transcript abundance results in significative inverse correlation relationship with *CHD8* both in the ASD patients group (R = −0.38, *p* = 0.0007) and in the healthy family members, i.e., the control group (R = –0.59, *p* = 1.63e-08).

In order to validate the transcriptional upregulation initially observed following *CHD8* suppression (Sugathan et al., 2014) (Table 1), *RAB11B/RAB11B-AS1* S/AS pair was quantified by qPCR in independent biological replicates of *CHD8* KD hiNPCs. Conforming to initial RNA-seq results (Table 1), *RAB11B* exhibited a mild upregulation (*p*-value = 0.06), while *RAB11B-AS1* dysregulation was more robust and significant in *CHD8*-suppressed lines (Figure 2A). We further calculated a linear regression analysis to appreciate possible correlation between the levels of *CHD8* KD and the expression of the S/AS pair. By resorting to the initial logCPM from the hiNPCs models with *CHD8* suppression (Sugathan et al., 2014), we uncovered a significant anti-correlation between *RAB11B-AS1* and *CHD8,* while *RAB11B* correspondence was milder (Figure 2B).

Next, to further dissect the expression crosstalk between *CHD8* and *RAB11B/RAB11B-AS1* S/AS pair we resorted to blood transcriptomic data of the Italian Autism Network (ITAN) (Muglia et al., 2018). RNA-seq data derived from peripheral blood samples of ASD and unaffected siblings (Filosi et al., 2020) were tested. While a modest (R_ASD_ = 0.036; R_NO ASD_ = −0.18) anti-correlation between *RAB11B* and *CHD8* was observed (Figure 2C, top), a significant inverse correlation between *RAB11B-AS1* and *CHD8* expression levels was found in both ASD and control siblings (Figure 2C, bottom). Altogether, these results suggest a possible functional suppression mechanism by *CHD8* on the *RAB11B/RAB11B-AS1* locus, which might be impaired in *CHD8* haploinsufficiency conditions.

### *RAB11B-AS1* Over-Expression is Able to Enhance RAB11B Translation With No Transcriptional Alteration

**FIGURE 3:**
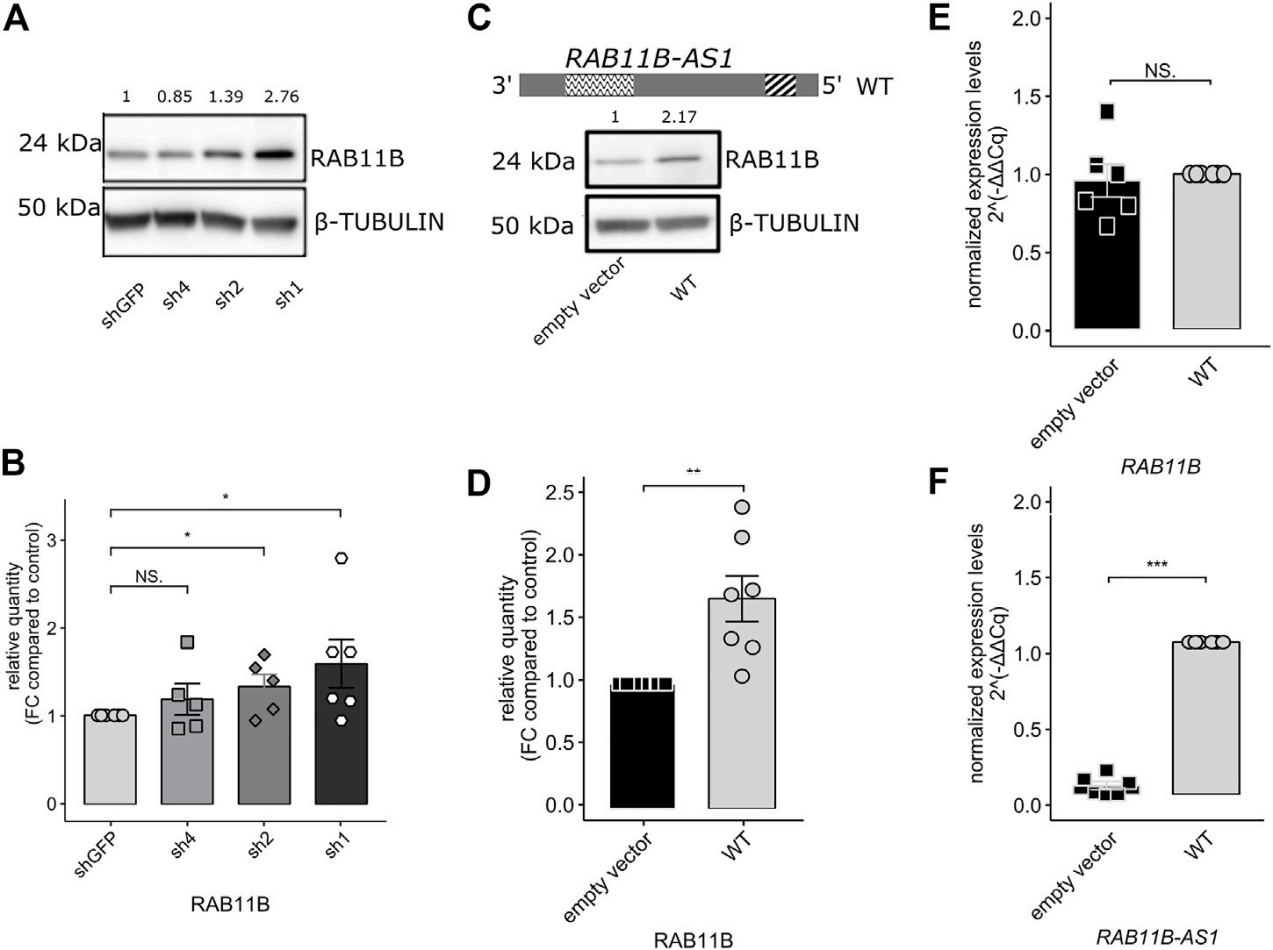
*RAB11B-AS1* (WT) over-expression leads to RAB11B protein stabilization without affecting its mRNA levels. **(A)** Representative Western Blot shows RAB11B levels increase in *CHD8* KD lines (sh4, sh2, sh1). β-TUBULIN was used as loading control. **(B)** The barplot reports the densitometric analysis of WB experiments. RAB11B protein levels increase in *CHD8* KD lines (significance in sh2 and sh1 lines). Data are plotted as Mean ± SEM. For statistical analysis, un-paired one-tailed Student’s *t*-test was performed. N = 5–6. NS *p* > 0.05, *p ≤ 0.05. **(C)** The *RAB11B-AS1* (WT) sequence schematic reports the location of the putative SINEUP domains **(up)**. Representative Western Blot image **(down)** depicts RAB11B protein increase upon *RAB11B-AS1* (WT) over-expression in GM8330-8 hiNPC line. β-TUBULIN was used as loading control. **(D)** Barplot of RAB11B densitometric analysis, confirming that protein increase was statistically significant upon antisense lncRNA over-expression. **(E)** The barplot reports *RAB11B* transcriptional levels, tested by qPCR analysis and expressed as 2^−∆∆Cq^. mRNA levels were stable upon *RAB11B-AS1* over-expression (black) with respect to the control empty vector (white). **(F)** *RAB11B-AS1* was abundantly and significantly overexpressed upon plasmid construct delivery (black) with respect to the control (white). Data are plotted as Mean ± SEM of N = 6 biological replicates. For statistical analysis, un-paired one-tailed Student’s *t*-test was performed. Sample size N = 6. NS *p* > 0.05, **p ≤ 0.01, ***p ≤ 0.001.

Because a measurable effect of a functional SINEUP molecule is the increase in translation of its sense counterpart and considering the over-expression of *RAB11B-AS* in the *CHD8* suppression lines, we predicted increased levels for RAB11B protein. After Western Blot quantification, densitometric analysis of the 24 kDa bands corresponding to RAB11B highlighted a significant increase in protein levels upon *CHD8* suppression with respect to the control condition (Figures 3A,B). While RAB11B upregulation was solid and reproducible in *CHD8*-Sh2 and *CHD8*-Sh1, displaying roughly 50% of *CHD8* KD, the data on the third KD line (*CHD8*-Sh4) seem to be more variable.

Finally, to functionally characterize *RAB11B-AS1* as a SINEUP molecule, the full-length (WT) sequence of the transcript (Figure 3C, top) was cloned and over-expressed in GM8330-8 hiNPC parental line. As a result of *RAB11B-AS1* over-expression, RAB11B protein level was increased by approximately two times compared to control (Figures 3C,D). RAB11B protein up-regulation was significant and reproducible, as confirmed by statistical analysis of replicate experiments (Figure 3D). Furthermore, qPCR experiments confirmed that, despite the RAB11B protein increase, *RAB11B* transcriptional levels were substantially stable (Figure 3E) while *RAB11B-AS1* was abundantly over-expressed (Figure 3F). These results strongly support our initial hypothesis, as they are coherent with the functional mechanism of a SINEUP molecule.

### RAB11B Translational Increase is Dependent on the Presence of *RAB11B-AS1* SINEUP Functional Domains

**FIGURE 4:**
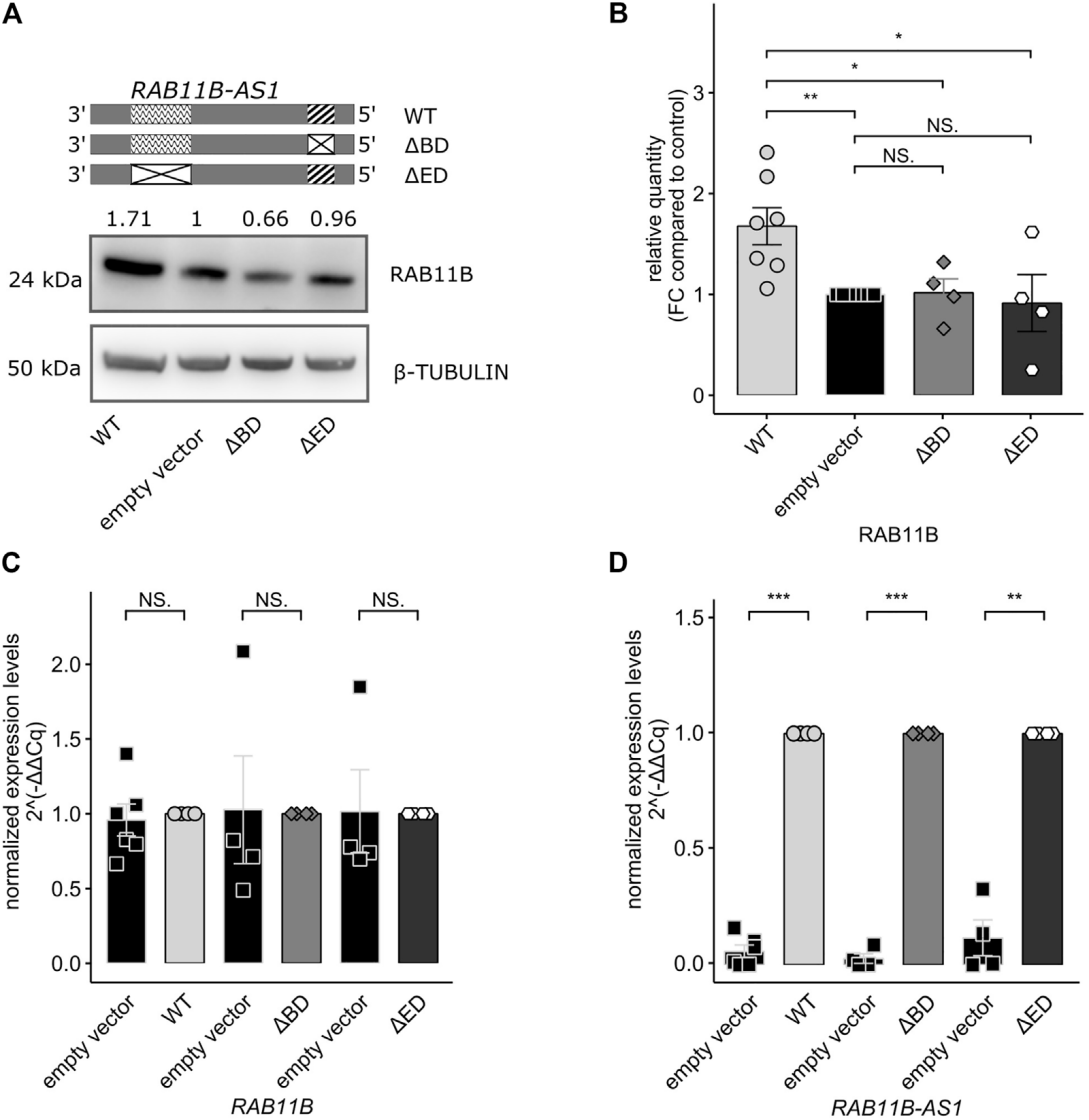
Deletion of functional SINEUP domains (BD, ED) impairs *RAB11B-AS1* effect on RAB11B protein translation. **(A)** The *RAB11B-AS1* (WT, ∆BD, and ∆ED) sequence schematics report the location of the putative SINEUP domains (up). Representative image describes a Western Blot of RAB11B after *RAB11B-AS1* WT, ∆BD, and ∆ED over-expression. β-TUBULIN was used as loading control. **(B)** Barplot of RAB11B densitometric analysis, confirming that protein increase was statistically significant upon over-expression of *RAB11B-AS1* (WT), while deletion mutants over-expression caused no protein expression difference from the control empty vector. **(C)** The barplot shows *RAB11B* transcriptional levels, tested by qPCR and expressed as 2^−∆∆Cq^. mRNA levels were stable upon plasmid constructs over-expression (WT = black, ∆BD = light grey, ∆ED = dark grey) with respect to the control empty vector (white). **(D)** *RAB11B-AS1* was abundantly and significantly overexpressed upon plasmid delivery (WT = black, ∆BD = light grey, ∆ED = dark grey) with respect to the control (white). Data are plotted as Mean ± SEM, N = 4 for deletion mutants, N = 6 for WT over-expression. For statistical analysis, un-paired one-tailed Student’s *t*-test was performed. NS *p* > 0.05, *p ≤ 0.05, **p ≤ 0.01, ***p ≤ 0.001.

In order to fully prove the SINEUP nature of *RAB11B-AS1* lncRNA, we wanted to test whether the absence of one of the putative functional domains might impair the SINEUP-like mechanism. To this purpose, two domain-specific deletion mutants were generated by site-specific mutagenesis (∆BD or ∆ED *RAB11B-AS1*). *RAB11B-AS1* WT, ∆BD or ∆ED were then delivered in parental GM8330-8 hiNPCs, and subsequently Western Blot experiments were performed to quantify RAB11B protein level. While the over-expression of WT, full-length *RAB11B-AS1* elicited the expected increase in RAB11B protein, ∆BD, and ∆ED mutants failed to evoke RAB11B protein upregulation, in line with the anticipated SINEUP activity (Figure 4A). Such observation was confirmed by densitometric analysis on replicated experiments (*n* = 4–6) (Figure 4B). Importantly, qPCR revealed that *RAB11B* transcriptional levels were stable (Figure 4C) while *RAB11B-AS1* WT and deletion mutants were significantly and strongly over-expressed (Figure 4D). These results suggest that RAB11B protein translation increase is mediated by its antisense transcript *RAB11B-AS1* functional domains. Taken together, these results are reinforcing the hypothesis that *RAB11B-AS1* lncRNA is a *CHD8-*suppression-sensitive SINEUP molecule, able to up-regulate protein translation of its target mRNA *RAB11B* and potentially relevant for *CHD8* haploinsufficiency defined ASD.

## Discussion

NcRNAs constitute the major product of mammalian transcription (The FANTOM Consortium, 2005), however their functions are still largely unexplored. Hinting at their possible role in higher cognition, lncRNAs are primarily expressed in the brain (Mercer et al., 2009), with definite patterns across cerebral areas, and several of them exclusively described in primates (Mattick, 2018). Increasing evidence underscores their role in neuronal physiology and pathology. In fact, lncRNAs have been implicated in neural development and functioning (Andersen and Lim, 2018), brain aging and neurodegeneration (Wan et al., 2017), but also neurodevelopmental disorders, such as ASD.

In this study, we sought to characterize the effects of transcriptional dysregulation of lncRNAs in a model system of neuronal development and relevant to ASD. Specifically, we resorted to hiNPCs, where *CHD8* expression was reduced by short-hairpins administration to roughly 50%. CHD8 protein haploinsufficiency represents one of the highest confidence risk factors for ASD with profound consequences for the whole transcriptome. In this work, we identified SINEUP-like antisense lncRNAs among the pool of dysregulated genes following *CHD8* suppression (Sugathan et al., 2014). SINEUP ncRNAs are a class of regulatory, antisense modular transcripts, which increase protein translation of their sense mRNA by means of their characteristic functional domains (Zucchelli et al., 2015a; Zucchelli et al., 2015b). Thus, we filtered, relying merely on structural features, the 1756 DEGs upon *CHD8* suppression (Sugathan et al., 2014). Only an exiguous list of candidates met our stringent criteria. However, we cannot exclude that the number of dysregulated SINEUP molecules might be underestimated in our study. In fact, due to the poly-A mRNA enrichment protocol used for library preparation (Sugathan et al., 2014), a large portion of poly-A-minus lncRNAs (Mattick, 2018), and possibly also non-polyadenylated SINEUP, might have been missed. A total of 5 SINEUP-like molecules have been identified (Table 1), however, we prioritized for further functional validation *RAB11B-AS1,* which displayed the structural organization more typically associated with natural SINEUP. Importantly, a murine ortholog of *RAB11B-AS1* was identified, with an inverted SINEB2 TE. LncRNAs containing embedded TEs are more conserved across species with respect to non-TE-derived sequences, and display significantly less variance (Kapusta et al., 2013), sustaining the hypothesis that TEs in lncRNAs are subject to functional and/or structural constraints during evolution. Previous reports in osteosarcoma, lung, and breast cancer development described different, discrepant modes of *RAB11B-AS1* regulation on *RAB11B* mRNA and protein levels: downregulation (Chen et al., 2018), upregulation (Li et al., 2020) or no effect (Niu et al., 2020) of the sense transcript was observed, generating an inconclusive scenario. Notably, *RAB11B*–the head-to-head protein coding transcript, overlapping *RAB11B-AS1*–has critical roles in apical recycling of cargo proteins (Delisle et al., 2009; Silvis et al., 2009; Best et al., 2011; Butterworth et al., 2012). Moreover, it was reported to inhibit Ca^2+^-triggered exocytosis in neuronal and neuroendocrine cells, and to be enriched in purified synaptic vesicles (Khvotchev et al., 2003). Importantly, *RAB11B de novo* mutations were correlated with Intellectual Disability and microcephaly (Lamers et al., 2017). Thus, these observations globally support a role for RAB11B–and possibly its overlapping lncRNA–in vesicular trafficking and synaptic activity, of relevance for ASD and other neurological conditions. Firstly, we validated the upregulation of *RAB11B*/*RAB11B-AS1* transcripts pair following *CHD8* suppression by qPCR. Secondly, we confirmed a comparable expression pattern between *RAB11B-AS1*/*RAB11B* across human body districts and CAGE data, coherently with previous observations reporting a similar spatio-temporal distribution of S/AS pairs (Chen et al., 2005). Furthermore, we uncovered an anti-correlation between *CHD8* and *RAB11B-AS1* in ASD-affected and healthy siblings of the ITAN cohort. Thus, aberrantly reduced expression of *CHD8* seems to correlate with *RAB11B-AS1* upregulation. However, in our hiNPCs model transcriptomic data, both *RAB11B-AS1* and *RAB11B* appear to be upregulated, although with different strength and significance. While further studies will be needed to fully dissect this interplay, the observed upregulation of the sense *RAB11B* transcript might be directly mediated by CHD8 protein, since CHD8 binding sites were identified on *RAB11B*, but not on *RAB11B-AS1* promoter (Sugathan et al., 2014).

Finally, we moved to the functional characterization of *RAB11B-AS1* as a potential new SINEUP molecule. To this task, we cloned and overexpressed the full-length human lncRNA transcript. Over-expression of *RAB11B-AS1* did not affect *RAB11B* transcriptional levels but led to a reproducible increase in the production of RAB11B protein. This post-transcriptional effect is consistent with a SINEUP role, as translation is typically expected to increase in the range of 1.5–3 fold (Zucchelli et al., 2015a). To further strengthen our results, we created deletion mutants of *RAB11B-AS1*, removing the SINEUP functional domains (BD and ED). Consistently with our hypothesis, the mutant forms of the transcript failed to exert a regulatory effect on *RAB11B* mRNA translation. Thus, here we propose that *RAB11B-AS1* SINEUP molecule potentially represents a further indirect layer of protein translation regulation, independent of *RAB11B* transcriptional control. This finding seems to be discordant with previous studies (Chen et al., 2018; Li et al., 2020; Niu et al., 2020), however, AS-lncRNAs have been previously reported to have dual functions, and this could depend on the cellular context and availability of specific co-factors. To this point, *Uxt-AS1*, initially found to act as a SINEUP by upregulating protein translation of its sense counterpart *Uxt* (Carrieri et al., 2012), in a later study was, instead, found to regulate alternative splicing of *UXT* in human colonic carcinoma cell lines (Yin et al., 2017). Thus, alternative roles for some lncRNAs could be described when using different cell lines or other cellular contexts or tissues. This could suggest that expression of different mediators could drive different functional effects of specific AS-lncRNA on their sense counterparts.

In conclusion, we provided evidence that naturally occurring SINEUP could be involved in ASD pathogenesis, highlighting the importance of dysregulation of functional lncRNAs during brain development.

## Data Availability

Publicly available datasets were analyzed in this study. This data can be found here: http://www.pnas.org/lookup/suppl/doi:10.1073/pnas.1405266111/-/DCSupplemental/pnas.1405266111.sd01.xlsx, “Sugathan A, Biagioli M, Golzio C, Erdin S et al. CHD8 regulates neurodevelopmental pathways associated with autism spectrum disorder in neural progenitors. Proc Natl Acad Sci U S A 2014 Oct 21;111 (42):E4468-77. PMID: 25294932”, GSE61491, GEO, NCBI; CAGE data were retrieved from ZENBU Severin J, Lizio M, Harshbarger J, Kawaji H, Daub CO, Hayashizaki Y; FANTOM Consortium, Bertin N, Forrest AR. Interactive visualization and analysis of large-scale sequencing datasets using ZENBU. Nat Biotechnol. 2014 Mar;32 (3):217-9. doi: 10.1038/nbt.2840. PMID: 24727769.
